# E-Cigarette Characteristics and Cigarette Cessation Among Adults Who Use E-Cigarettes

**DOI:** 10.1001/jamanetworkopen.2024.23960

**Published:** 2024-08-01

**Authors:** Karin A. Kasza, Cheryl Rivard, Maciej L. Goniewicz, Geoffrey T. Fong, David Hammond, K. Michael Cummings, Andrew Hyland

**Affiliations:** 1 Department of Health Behavior, Roswell Park Comprehensive Cancer Center, Buffalo, New York; 2 Department of Psychology, University of Waterloo, Waterloo, Ontario, Canada; 3 Ontario Institute for Cancer Research, Toronto, Ontario, Canada; 4 School of Public Health Sciences, University of Waterloo, Waterloo, Ontario, Canada; 5 Department of Psychiatry and Behavioral Sciences, Medical University of South Carolina, Charleston, South Carolina

## Abstract

**Importance:**

Population-level health outcomes associated with e-cigarettes depend in part on the association between e-cigarettes and combustible cigarette cessation. The US Food and Drug Administration has authority to regulate e-cigarette characteristics, including flavor and device type.

**Objective:**

To investigate whether e-cigarette characteristics are associated with cigarette cessation behaviors among adults in the US population who use e-cigarettes.

**Design, Setting, and Participants:**

This cohort study was conducted using longitudinal data collected in 2014 to 2021 by the Population Assessment of Tobacco and Health Study, a population-based, US nationally representative study. Participants were sampled from the civilian noninstitutionalized population using a 4-staged, stratified sampling design. Data were weighted and analyzed from 1985 adults ages 21 or older who smoked cigarettes daily and had used e-cigarettes in the past 30 days. Data were analyzed in May 2021 to May 2024.

**Exposures:**

The following e-cigarette characteristics were assessed: use frequency (daily and nondaily), flavor type (tobacco, menthol or mint, sweet, and combination), device type (disposable, cartridge, and tank), and year of data collection as a proxy for the evolving e-cigarette marketplace.

**Main Outcomes and Measures:**

The following cigarette cessation behaviors were assessed: making a cigarette quit attempt, cigarette cessation among individuals who made a quit attempt, and overall cigarette discontinuation regardless of quit attempts. Associations were evaluated between e-cigarette characteristics (assessed at baseline in 1 approach and assessed at follow-up in another approach) and cigarette cessation outcomes, controlling for demographic, cigarette smoking, and other e-cigarette use characteristics.

**Results:**

The study sample consisted of 1985 participants representing adults in the population (mean age, 40.0 years [95% CI, 39.2-40.9 years]; 49.4% [95% CI, 46.3%-52.6%] male; 11.4% [95% CI, 9.6%-13.4%] Black, 80.7% [95% CI, 77.8%-83.3%] White, and 8.0% [95% CI, 6.3%-10.0%] other race; 9.2% [95% CI, 7.5%-11.2%] Hispanic). Daily vs nondaily e-cigarette use was associated with greater overall cigarette discontinuation rates (12.8% [95% CI, 9.1%-17.7%] vs 6.1% [95% CI, 4.8%-7.7%]; adjusted odds ratio [aOR], 2.26 [95% CI, 1.34-3.81]), and use of e-cigarettes in 2019 to 2021 vs 2014-2015 to 2015-2016 was also associated with greater overall cigarette discontinuation rates (12.0% [95% CI, 8.8%-16.0%] vs 5.3% [95% CI, 2.9%-9.3%]; aOR, 2.75 [95% CI, 1.13-6.67]). Use of menthol or mint vs tobacco flavor e-cigarettes was associated with greater overall cigarette discontinuation rates (9.2% [95% CI, 6.6%-12.8%] vs 4.7% [95% CI, 3.0%-7.1%]; aOR, 2.63 [95% CI, 1.32-5.27]) only when assessing e-cigarette use at baseline. E-cigarette device type was not associated with cigarette discontinuation rates in adjusted analyses.

**Conclusions and Relevance:**

In this study, daily e-cigarette use and use of e-cigarettes in 2019 to 2021 were consistently associated with greater cigarette discontinuation rates. These findings suggest that research focused on e-cigarettes marketed in recent years is needed to inform product regulation and public health policy decisions.

## Key Points

**Question:**

Are e-cigarette characteristics associated with cigarette cessation behaviors among adults in the US population who smoke cigarettes and use e-cigarettes?

**Findings:**

In this cohort study of 1985 adults who smoked cigarettes and used e-cigarettes, use of e-cigarettes in 2019 to 2021 vs 2014-2015 to 2015-2016 and daily use of e-cigarettes vs nondaily use were associated with greater overall cigarette discontinuation rates.

**Meaning:**

These results suggest that public health policy decisions regarding e-cigarettes should be based on data from e-cigarettes marketed in recent years that have evolved outside the regulated market.

## Teaser

This cohort study uses data from the Population Assessment of Tobacco and Health (PATH) Study to investigate the association of e-cigarette characteristics with cigarette cessation behaviors among adults who use e-cigarettes.

## Introduction

Cigarettes are overwhelmingly responsible for the burden of death and disease caused by tobacco.^1^ As such, the association of electronic nicotine delivery systems (hereafter, *e-cigarettes*) with combustible tobacco cigarette smoking is a key contributor to the population-level outcomes associated with e-cigarettes. In 2018, the National Academies of Sciences, Engineering, and Medicine concluded that frequent use of e-cigarettes among people who smoke cigarettes was associated with greater cigarette cessation.^2^ Similarly, a subsequent meta-analysis by Wang et al^3^ concluded that daily e-cigarette use was associated with greater cigarette cessation compared with nondaily use or no e-cigarette use and that nondaily e-cigarette use was associated with lower cigarette cessation compared with no e-cigarette use. From a nicotine dependence standpoint, higher-frequency e-cigarette use may provide more nicotine to the consumer, which in turn may increase the substitution potential of e-cigarettes for cigarettes.^2^ However, it is also possible that higher-frequency e-cigarette use among people who smoke cigarettes is itself an indicator of greater motivation or commitment to quit smoking.^4^ That is, e-cigarette use frequency may be associated with motivational and effectiveness components of the cigarette cessation process.

E-cigarette design features, such as flavor and device type, can be regulated in the US by the Food and Drug Administration (FDA) Center for Tobacco Products (CTP).^5,6^ Therefore, determining whether such features of e-cigarette use (necessarily among individuals who use e-cigarettes) are associated with cigarette cessation behaviors is important to informing tobacco regulatory decisions. A 2023 systematic review^7^ evaluated the association of e-cigarette flavors with cigarette cessation outcomes and determined the literature in this area to be inconclusive owing to a paucity of research, heterogeneity in definitions, and limitations in methods used.

Given the established importance of e-cigarette use frequency in cigarette smoking cessation behaviors,^2,3,4^ it is important to understand associations of e-cigarette flavor types and device types with smoking cessation independent of any association these features may have with e-cigarette use frequency. Indeed, using US nationally representative data from the Population Assessment of Tobacco and Health (PATH) Study, Kasza et al^8^ found that e-cigarette flavor and e-cigarette device type were each associated with e-cigarette use frequency among adults who used e-cigarettes and attempted to quit cigarette smoking; those who used sweet flavor e-cigarettes had higher use frequency than those who used tobacco flavor e-cigarettes, and those who used tank-style e-cigarettes had higher use frequency than those who used cartridge or disposable e-cigarettes. If use of sweet flavor, tank device types, or both are associated with greater cigarette cessation than use of other flavor and device types, then e-cigarette use frequency could be the mechanism underlying the association, which could explain why flavor and device type may be important in cigarette cessation.

Several studies have evaluated the association of e-cigarette use vs no e-cigarette use with cigarette cessation in the population^9,10,11^ and have generally found daily e-cigarette use to be positively associated with cigarette cessation and nondaily e-cigarette use to be negatively associated with cigarette cessation.^3^ The purpose of this study was to evaluate in the US adult population of people who smoke cigarettes and use e-cigarettes whether there were associations between e-cigarette characteristics and cigarette cessation behaviors. Specifically, among adults who used e-cigarettes and smoked cigarettes daily, we evaluated associations of e-cigarette use frequency, e-cigarette flavor, e-cigarette device type, and year of data collection (a proxy for the evolving e-cigarette marketplace) with attempting to quit cigarette smoking, cigarette cessation among individuals who attempted to quit, and cigarette discontinuation (ie, the overall transition to no cigarette smoking regardless of making a quit attempt).

## Methods

The PATH Study was conducted by Westat and approved by the Westat Institutional Review Board. This cohort study was approved by the Roswell Park Institutional Review Board. All adults provided written informed consent, which extends to the current study. We followed the Strengthening the Reporting of Observational Studies in Epidemiology (STROBE) reporting guideline for cohort studies.

### Participants

We analyzed data from adults who participated in the PATH Study, a nationally representative longitudinal study of youth and adults in the US.^12,13^ Data were collected using audio, computer-assisted self-interviews conducted in English or Spanish from October 2014 to October 2015 (wave 2; hereafter, *2014-2015*), October 2015 to October 2016 (wave 3; 2015-2016), December 2016 to January 2018 (wave 4; 2017), and December 2018 to November 2019 (wave 5; 2019). Due to the COVID-19 pandemic, data were collected using both audio computer-assisted self-interviews and telephone interviews from March to November 2021 (wave 6; 2021).^12^

Response rates for adults ranged from 58% in wave 6 to 83% in wave 2.^12^ Additional details on the PATH Study design and methods^14,15,16^ and demographic and tobacco use distributions^17^ are published elsewhere. Details on interviewing procedures, questionnaires, sampling, weighting, response rates, nonresponse bias analysis reports, and accessing the data are available at the National Addiction and HIV Archive Program.^12,18^

We conducted analyses among 1985 adults ages 21 years or older (in accordance with Tobacco 21 laws) who smoked cigarettes daily at the baseline wave of a wave pair (ie, wave 2 in the wave 2–wave 3 pair, wave 3 in the wave 3–wave 4 pair, wave 4 in the wave 4–wave 5 pair, and wave 5 in the wave 5–wave 6 pair) and who had used e-cigarettes in the past 30 days at the baseline wave of the wave pair. That is, we evaluated 4 time intervals, each including 2 waves consisting of a baseline wave and a follow-up wave. We further describe this approach and an additional approach to analyses in the eMethods, eFigure 1, and eFigure 2 in Supplement 1.

### Measures

#### E-Cigarette Characteristics

At each interview, respondents were asked whether they used e-cigarettes every day, some days, or not at all, and those who used e-cigarettes were asked, “What flavor is [your regular brand/the brand you last used]? Choose all that apply.” Response options were tobacco flavored (except in wave 2, where “tobacco flavor” was not listed, so respondents who reported not using a flavor in wave 2 were coded as using tobacco flavor); menthol or mint; clove or spice; fruit; chocolate; an alcoholic drink (such as wine, cognac, or margarita or other cocktails), a nonalcoholic drink (such as coffee, soda, energy drinks, or other beverages); candy, desserts, or other sweets; and some other flavor. We categorized e-cigarette flavor type into 4 mutually exclusive categories to broadly align with US regulatory approaches: only tobacco flavor; only menthol or mint flavor; only nontobacco, nonmenthol and nonmint flavor (including any flavor except tobacco or menthol or mint flavor; hereafter, *sweet*); and any combination of tobacco, menthol or mint, sweet flavors, or unflavored.

Respondents who used e-cigarettes were also asked if the e-cigarette product they used most often was rechargeable (yes or no) and whether it used cartridges (yes or no). We categorized e-cigarette device type into 3 mutually exclusive categories: disposable product that is not rechargeable, cartridge or pod device that is rechargeable and uses prefilled cartridges or pods, and tank device that is rechargeable and does not use prefilled cartridges or pods.

Lastly, we considered year of data collection as a proxy for the evolving e-cigarette marketplace. Year of data collection was evaluated in analyses using wave pairs as follows: 2014-2015 to 2015-2016, 2015-2016 to 2017, 2017 to 2019, and 2019 to 2021.

#### Cigarette Cessation Behaviors

At the follow-up wave of each wave pair, respondents who currently smoked cigarettes were asked, “In the past 12 months, have you tried to quit [cigarettes/tobacco] completely?” Respondents who smoked cigarettes and used other tobacco products were asked about quitting “tobacco.” Among respondents who smoked daily at baseline, we defined making a quit attempt at follow-up as having tried to quit using cigarettes or tobacco completely in the past 12 months or currently not smoking cigarettes at all. Among respondents who smoked daily at baseline and reported making a quit attempt at follow-up, we defined cigarette cessation as no smoking at all at follow-up. Lastly, among respondents who smoked daily at baseline, we defined cigarette discontinuation as the overall transition to no past 30-day smoking at follow-up regardless of making a quit attempt.

#### Other Measures

Respondents self-reported their age, biological sex (male or female), race (Black, White, other race [American Indian or Alaska Native, Asian Indian, Chinese, Filipino, Japanese, Korean, Vietnamese, Other Asian, Native Hawaiian, Guamanian or Chamorro, Samoan, and Other Pacific Islander]), ethnicity (not of Hispanic [Latino/Latina/Latino or Latina] or Spanish origin; Mexican, Mexican American, [Chicano/Chicana/Chicano or Chicana]; Puerto Rican; Cuban; another Hispanic [Latino/Latina/Latino or Latina] or Spanish origin), cigarettes smoked per day at baseline, and cigarette flavor smoked at baseline (menthol or nonmenthol based on whether any cigarettes smoked in the past 30 days were menthol cigarettes). Each variable and interview mode (in person or telephone, as described previously) was included as a covariate in adjusted analyses.

### Statistical Analysis

We conducted analyses using the 2 approaches to ascertaining the e-cigarette use sample indicated previously: respondents who used e-cigarettes at baseline when they were also smoking cigarettes (eFigure 1 in Supplement 1) and those who used e-cigarettes at follow-up when the cigarette cessation outcome was assessed (eFigure 2 in Supplement 1). For each approach, we determined cigarette cessation behavior rates at follow-up stratified by e-cigarette use frequency, e-cigarette flavor, e-cigarette device type, and year of data collection.

Next, we used generalized estimating equations (GEEs) for each approach to evaluate associations of e-cigarette use frequency, e-cigarette flavor, e-cigarette device type, and year of data collection with each cigarette cessation behavior outcome. GEE analyses were conducted using 4 wave pairs: 2014-2015 to 2015-2016, 2015-2016 to 2017, 2017 to 2019, and 2019 to 2021. This allowed for the assessment of change in cigarette smoking between baseline and follow-up from all wave pairs in a single analysis while statistically controlling for interdependence among observations contributed by the same individuals.^19,20^ This approach allows for a robust evaluation of meaningful transitions in cigarette smoking behaviors over relatively shorter periods of time rather than evaluating numerous trajectories in product use over longer periods across all the waves of data. GEE logistic regression models specified unstructured covariance and within-person correlation matrices and a binomial distribution of the dependent variable using the logit link function. A 2-sided α of .05 was considered statistically significant. Analyses were adjusted for sex, race, ethnicity, age, cigarettes smoked per day, menthol or nonmenthol cigarette smoking, e-cigarette flavor, e-cigarette device type, interview mode, and year of data collection (model 1). In a second model, analyses were adjusted for the same covariates as in model 1 plus e-cigarette use frequency to determine whether any associations of e-cigarette flavor, device type, or year of data collection with cigarette cessation outcomes were mitigated by e-cigarette use frequency (model 2).

Missing data on age and sex were imputed by the PATH Study. Respondents who were missing data on race were grouped with the “Other race, including multiple races” category. Respondents who reported that they did not know whether they smoked menthol or nonmenthol cigarettes were treated as a valid unknown category. Respondents who were missing data on any other variables were excluded from analyses using listwise deletion. All analyses were weighted to adjust for the PATH Study’s complex study design characteristics (eg, oversampling) and attrition to produce estimates that were nationally representative for each year of data collection, and variances were computed using the balanced repeated replication method^21^ with Fay adjustment set to 0.3.^22^ Analyses were conducted using Stata statistical software version 17.0 (StataCorp), adapting SAS macro code to Stata code to run weighted GEE analyses and calculate adjusted odds ratios (aORs) and CIs.^23^ Analyses were run on the W2 to W6 restricted use files.^12^ Data were analyzed May 2021 to May 2024.

## Results

The composition of the population of 1985 adults (mean age, 40.0 years [95% CI, 39.2-40.9 years]; 49.4% [95% CI, 46.3%-52.6%] male; 11.4% [95% CI, 9.6%-13.4%] Black, 80.7% [95% CI, 77.8%-83.3%] White, and 8.0% [95% CI, 6.3%-10.0%] other race; 9.2% [95% CI, 7.5%-11.2%] Hispanic) ages 21 years or older who were included in our main analyses (e-cigarette use assessed prior to the outcome) are shown in Table 1 (weighted percentages). Please see eTable 1 in Supplement 1 for a description of the population included in our second approach to analyses (e-cigarette use assessed contemporaneously with the outcome). We report on results among adults who smoked cigarettes and used e-cigarettes at baseline (ie, first approach as described previously and shown in eFigure 1 in Supplement 1) in Table 2, Table 3, and Table 4. We report on results among adults who smoked cigarettes at baseline and used e-cigarettes at follow-up (ie, the second approach to analyses described in eMethods and shown in eFigure 2 in Supplement 1) in eTables 2 to 4 in Supplement 1.

**Table 1.** Population Characteristics

Characteristic	Participants, No. (N = 1985)^a^	% (95% CI)^a^
Age, mean, y	1985	40.0 (39.2-40.9)
Sex		
Male	823	49.4 (46.3-52.6)
Female	1162	50.6 (47.4-53.7)
Race		
Black	263	11.4 (9.6-13.4)
White	1520	80.7 (77.8-83.3)
Other race including multiple races^b^	202	8.0 (6.3-10.0)
Ethnicity		
Hispanic	214	9.2 (7.5-11.2)
Non-Hispanic	1771	90.8 (88.8-92.5)
CPD		
1-9	551	25.0 (22.5-27.7)
10-19	733	36.0 (33.0-39.1)
≥20	701	38.9 (35.9-42.0)
Flavored cigarette smoking		
Menthol	974	46.8 (43.8-49.8)
Nonmenthol	1000	52.8 (49.8-55.7)
Unknown or missing	11	0.4 (0.2-0.9)
E-cigarette use frequency		
Daily	283	15.6 (13.5-18.0)
Nondaily	1702	84.4 (82.0-86.5)
E-cigarette device type used		
Disposable	319	15.8 (13.8-18.0)
Cartridge	764	38.9 (36.4-41.5)
Tank	902	45.3 (42.4-48.3)
E-cigarette flavor used		
Only tobacco	512	27.9 (24.9-31.2)
Only menthol or mint	388	18.8 (16.6-21.2)
Only sweet flavor	878	42.9 (39.5-46.4)
Any combination of tobacco, menthol or mint, and sweet flavor	207	10.4 (8.6-12.5)
Year of data collection		
2014-2015 to 2015-2016 (W2-W3)	288	15.3 (13.5-17.3)
2015-2016 to 2017 (W3-W4)	584	30.2 (28.4-32.1)
2017-2019 (W4-W5)	501	24.8 (23.0-26.7)
2019-2021 (W5-W6)	612	29.7 (27.9-31.6)

Abbreviations: CPD, cigarettes smoked per day; W, wave.

^a^ Sample sizes are unweighted; percentages and 95% CIs are weighted.

^b^ Other race including multiple races includes American Indian or Alaska Native, Asian Indian, Chinese, Filipino, Japanese, Korean, Vietnamese, Other Asian, Native Hawaiian, Guamanian or Chamorro, Samoan, and Other Pacific Islander.

**Table 2.** Association of E-Cigarette Characteristics With Cigarette Quit Attempts

Characteristic (n = 1970)^a^	Cigarette quit attempt at follow-up^b^			
	Participants, No.	% (95% CI)	aOR (95% CI)	
			Model 1^c^	Model 2^d^
Frequency of e-cigarette use				
Nondaily (n = 1687)	699	40.6 (37.8-43.5)	NA	1 [Reference]
Daily (n = 283)	125	41.6 (34.1-49.6)		0.98 (0.68-1.40)
Device type used				
Disposable (n = 314)	125	39.9 (33.7-46.4)	1 [Reference]	1[Reference]
Cartridge (n = 758)	331	42.8 (38.1-47.7)	1.16 (0.78-1.74)	1.16 (0.78-1.74)
Tank or mod (n = 898)	368	39.4 (35.6-43.3)	1.04 (0.68-1.61)	1.05 (0.68-1.61)
Flavor used				
Only tobacco (n = 505)	202	38.2 (34.1-42.6)	1 [Reference]	1 [Reference]
Only menthol or mint (n = 383)	171	45.4 (39.8-51.2)	1.22 (0.83-1.80	1.22 (0.82-1.80)
Only sweet (n = 876)	363	40.0 (36.6-43.5)	0.95 (0.71-1.29)	0.95 (0.70-1.29)
Any combination of tobacco, menthol or mint, and sweet flavor (n = 206)	88	42.8 (34.9-50.8)	1.08 (0.74-1.58)	1.08 (0.74-1.58)
Year of data collection				
2014-2015 to 2015-2016 (W2-W3) (n = 287)	130	46.7 (39.7-53.8)	1 [Reference]	1 [Reference]
2015-2016 to 2017 (W3-W4) (n = 574)	216	36.5 (32.3-41.0)	0.69 (0.47-1.00)	0.68 (0.47-0.99)
2017 to 2019 (W4-W5) (n = 498)	222	42.1 (37.8-46.5)	0.88 (0.63-1.24)	0.88 (0.63-1.24)
2019 to 2021 (W5-W6) (n = 611)	256	41.0 (36.6-45.5)	0.79 (0.52-1.21)	0.79 (0.51-1.22)

Abbreviations: aOR, adjusted odds ratio; NA, not applicable; W, wave.

^a^ Characteristics are among adults who smoked cigarettes daily at baseline and used e-cigarettes in the past 30 days at baseline.

^b^ A quit attempt was defined as daily smoking at baseline with a quit attempt made at follow-up. Numbers indicate unweighted numbers of observations. Percentages, aORs, and 95% CIs are weighted using W6 all-waves weights for the W1 cohort. Generalized estimating equation logistic regression analyses were used to assess associations between e-cigarette characteristics and making a cigarette quit attempt using 4 wave pairs (W2-W3, W3-W4, W4-W5, and W5-W6), including up to 4 change data points per individual and statistically controlling for the correlation among observations from the same individuals.

^c^ Model 1 adjusts for sex, race, ethnicity, age, cigarettes smoked per day, menthol or nonmenthol cigarette smoking, interview mode, e-cigarette device type (except when evaluating the main association of e-cigarette device type used), e-cigarette flavor (except when evaluating the main association of e-cigarette flavor used), and year of data collection (except when evaluating the main association of year of data collection).

^d^ Model 2 adjusts for the same covariates as model 1 plus e-cigarette use frequency (except when evaluating the main association of e-cigarette use frequency).

**Table 3.** Association of E-Cigarette Characteristics With Cigarette Cessation Among Adults Who Made a Quit Attempt

Characteristic (n = 824)^a^	Cigarette cessation at follow-up^b^			
	Participants, No.	% (95% CI)	aOR (95% CI)	
			Model 1^c^	Model 2^d^
Frequency of e-cigarette use				
Nondaily (n = 699)	149	20.8 (17.4-24.6)	NA	1 [Reference]
Daily (n = 125)	52	39.1 (30.1-49.0)		2.44 (1.43-4.17)
Device type used				
Disposable (n = 125)	26	18.2 (12.8-25.3)	1 [Reference]	1 [Reference]
Cartridge (n = 331)	85	27.6 (22.1-34.0)	1.64 (0.99-2.72)	1.57 (0.94-2.62)
Tank or mod (n = 368)	90	22.0 (17.0-28.0)	1.02 (0.54-1.93)	0.92 (0.48-1.76)
Flavor used				
Only tobacco (n = 202)	37	18.9 (13.2-26.4)	1 [Reference]	1 [Reference]
Only menthol or mint (n = 171)	43	26.4 (19.1-35.3)	1.74 (0.91-3.32)	1.82 (0.95-3.49)
Only sweet (n = 363)	99	26.5 (21.5-32.2)	1.48 (0.83-2.63)	1.47 (0.81-2.64)
Any combination of tobacco, menthol or mint, and sweet (n = 88)	22	19.2 (11.4-30.5)	1.21 (0.55-2.66)	1.13 (0.48-2.65)
Year of data collection				
2014-2015 to 2015-2016 (W2-W3) (n = 130)	24	16.6 (10.6-25.1)	1 [Reference]	1 [Reference]
2015-2016 to 2017 (W3-W4) (n = 216)	40	16.6 (11.8-22.7)	1.10 (0.53-2.27)	1.26 (0.60-2.61)
2017 to 2019 (W4-W5) (n = 222)	47	22.2 (16.4-29.5)	1.62 (0.78-3.38)	1.72 (0.83-3.54)
2019 to 2021 (W5-W6) (n = 256)	90	35.6 (27.8-44.2)	3.24 (1.34-7.83)	3.32 (1.36-8.10)

Abbreviations: aOR, adjusted odds ratio; NA, not applicable; W, wave.

^a^ Characteristics are among adults who smoked cigarettes daily at baseline, used e-cigarettes in the past 30 days at baseline, and made a cigarette quit attempt between baseline and follow-up. Numbers indicate unweighted numbers of observations. Percentages, aORs, and 95% CIs are weighted using W6 all-waves weights for the W1 cohort. Generalized estimating equation logistic regression analyses were used to assess associations between e-cigarette use characteristics and cigarette cessation among adults who made a quit attempt using 4 wave pairs (W2-W3, W3-W4, W4-W5, and W5-W6), including up to 4 change data points per individual and statistically controlling for the correlation among observations from the same individuals.

^b^ Cigarette cessation was defined as daily smoking at baseline with a quit attempt between baseline and follow-up and no daily or nondaily smoking at follow-up.

^c^ Model 1 adjusts for sex, race, ethnicity, age, cigarettes smoked per day, menthol or nonmenthol cigarette smoking, interview mode, e-cigarette device type (except when evaluating the main association of e-cigarette device type used), e-cigarette flavor (except when evaluating the main association of e-cigarette flavor used), and year of data collection (except when evaluating the main association of year of data collection).

^d^ Model 2 adjusts for the same covariates as model 1 plus e-cigarette use frequency (except when evaluating the main association of e-cigarette use frequency).

**Table 4.** Association of E-Cigarette Characteristics With Discontinuing Past 30-d Cigarette Smoking

Characteristic (N = 1985)^a^	Discontinued cigarette smoking at follow-up^b^			
	Participants, No.	% (95% CI)	aOR (95% CI)	
			Model 1^c^	Model 2^d^
Frequency of e-cigarette use				
Nondaily (n = 1702)	109	6.1 (4.8-7.7)	NA	1 [Reference]
Daily (n = 283)	40	12.8 (9.1-17.7)		2.26 (1.34-3.81)
Device type used				
Disposable (n = 319)	20	5.8 (3.6-9.1)	1 [Reference]	1 [Reference]
Cartridge (n = 764)	64	8.8 (6.5-11.8)	1.38 (0.74-2.58)	1.33 (0.72-2.46)
Tank or mod (n = 902)	65	6.3 (4.7-8.4)	0.83 (0.44-1.58)	0.74 (0.39-1.40)
Flavor used				
Only tobacco (n = 512)	23	4.7 (3.0-7.1)	1 [Reference]	1 [Reference]
Only menthol or mint (n = 388)	35	9.2 (6.6-12.8)	2.60 (1.31-5.20)	2.63 (1.32-5.27)
Only sweet (n = 878)	72	8.0 (6.1-10.3)	1.78 (0.94-3.38)	1.78 (0.93-3.38)
Any combination of tobacco, menthol or mint, and sweet (n = 207)	19	7.0 (4.1-11.8)	1.79 (0.76-4.23)	1.77 (0.75-4.17)
Year of data collection				
2014-2015 to 2015-2016 (W2-W3) (n = 288)	15	5.3 (2.9-9.3)	1 [Reference]	1 [Reference]
2015-2016 to 2017 (W3-W4) (n = 584)	24	3.6 (2.4-5.5)	0.69 (0.30-1.60)	0.74 (0.32-1.70)
2017 to 2019 (W4-W5) (n = 501)	36	6.9 (4.7-10.1)	1.45 (0.63-3.31)	1.51 (0.66-3.48)
2019 to 2021 (W5-W6) (n = 612)	74	12.0 (8.8-16.0)	2.62 (1.08-6.37)	2.75 (1.13-6.67)

Abbreviations: aOR, adjusted odds ratio; NA, not applicable; W, wave.

^a^ Characteristics are among adults who smoked cigarettes daily at baseline and used e-cigarettes in the past 30 days at baseline. Numbers indicate unweighted numbers of observations. Percentages, aORs, and 95% CIs are weighted using W6 all-waves weights for the W1 cohort. Generalized estimating equation logistic regression analyses were used to assess associations between e-cigarette use characteristics and discontinuing cigarette smoking using 4 wave pairs (W2-W3, W3-W4, W4-W5, and W5-W6), including up to 4 change data points per individual and statistically controlling for the correlation among observations from the same individuals.

^b^ Discontinued cigarette smoking was defined as daily smoking at baseline to no past 30-day smoking at follow-up.

^c^ Model 1 adjusts for sex, race, ethnicity, age, cigarettes smoked per day, menthol or nonmenthol cigarette smoking, interview mode, e-cigarette device type (except when evaluating the main association of e-cigarette device type used), e-cigarette flavor (except when evaluating the main association of e-cigarette flavor used), and year of data collection (except when evaluating the main association of year of data collection).

^d^ Model 2 adjusts for the same covariates as model 1 plus e-cigarette use frequency (except when evaluating the main association of e-cigarette use frequency).

### Making a Cigarette Quit Attempt

Table 2 shows associations between e-cigarette characteristics assessed prior to the outcome and making a cigarette quit attempt among adults who had smoked cigarettes daily and used e-cigarettes in the past 30 days. There were no differences in making a cigarette quit attempt by e-cigarette use frequency, flavor, device type, or year of data collection with or without adjustment for e-cigarette use frequency (Table 2).

Associations between e-cigarette characteristics assessed contemporaneously with the outcome and making a cigarette quit attempt among respondents who had smoked cigarettes daily are shown in eTable 2 in Supplement 1. E-cigarette use frequency was associated with making a quit attempt (aOR for daily vs nondaily use, 3.12 [95% CI, 2.42-4.02]) (eTable 2 in Supplement 1). None of e-cigarette flavor, device type, or year of data collection were associated with making a quit attempt after adjusting for all covariates, including e-cigarette use frequency (eTable 2 in Supplement 1).

### Cigarette Cessation Among Adults Who Made a Cigarette Quit Attempt

Among adults who used e-cigarettes at baseline and made a cigarette quit attempt between baseline and follow-up, e-cigarette use frequency assessed prior to the outcome was associated with cigarette cessation at follow-up; in model 2, the cessation rate was 39.1% (95% CI, 30.1%-49.0%) for those who used e-cigarettes daily and attempted to quit compared with 20.8% (95% CI, 17.4%-24.6%) for those who used e-cigarettes nondaily and attempted to quit (aOR, 2.44 [95% CI, 1.43-4.17]) (Table 3). There was also an association for year of data collection in model 2; the cigarette cessation rate among adults who attempted to quit was 35.6% (95% CI, 27.8%-44.2%) in 2019 to 2021 compared with 16.6% (95% CI, 10.6%-25.1%) in 2014-2015 to 2015-2016 (aOR, 3.32 [95% CI, 1.36-8.10]) (Table 3). There were no differences in cigarette cessation by e-cigarette flavor or device type used at baseline among adults who attempted to quit (Table 3).

Among adults who attempted to quit, e-cigarette use frequency and year of data collection assessed contemporaneously with the outcome were each also associated with cigarette cessation (eTable 3 in Supplement 1). Neither e-cigarette flavor nor device type were associated with cigarette cessation among adults who attempted to quit (eTable 3 in the Supplement 1).

### Cigarette Discontinuation Overall Regardless of Making a Quit Attempt

E-cigarette use frequency assessed prior to the outcome was associated with overall cigarette discontinuation at follow-up in model 2; the cigarette discontinuation rate was 12.8% (95% CI, 9.1%-17.7%) for adults who used e-cigarettes daily compared with 6.1% (95% CI, 4.8%-7.7%) for those who used e-cigarettes nondaily (aOR, 2.26 [95% CI, 1.34-3.81]) (Table 4). There was also an association for year of data collection in model 2; the cigarette discontinuation rate was 12.0% (95% CI, 8.8%-16.0%) in 2019 to 2021 compared with 5.3% (95% CI, 2.9%-9.3%) in 2014-2015 to 2015-2016 (aOR, 2.75 [95% CI, 1.13-6.67]) (Table 4). Additionally, adults who used menthol or mint flavor e-cigarettes at baseline had higher rates of discontinuing cigarette smoking at follow-up (9.2% [95% CI, 6.6%-12.8%]) than those who used tobacco flavor e-cigarettes at baseline (4.7% [95% CI, 3.0%-7.1%) even after adjusting for all covariates, including e-cigarette use frequency, in model 2 (aOR, 2.63 [95% CI, 1.32-5.27]) (Table 4).

E-cigarette use frequency and year of data collection assessed contemporaneously with the outcome were each also associated with cigarette discontinuation (eTable 4 in Supplement 1). However, unlike e-cigarette flavor use assessed prior to the outcome, e-cigarette flavor use assessed contemporaneously with the outcome was not associated with cigarette discontinuation after adjusting for all covariates, including e-cigarette use frequency (eTable 4 in Supplement 1).

## Discussion

This cohort study’s nationally representative findings show that daily use of e-cigarettes compared with nondaily use of e-cigarettes was consistently associated with greater cigarette cessation rates among adults who used e-cigarettes and attempted to quit smoking and with greater overall cigarette discontinuation rates, consistent with prior literature,^2,3,4^ although daily use of e-cigarettes was uncommon. Furthermore, these findings align with research indicating that greater nicotine consumption from e-cigarette use increases the substitution potential of e-cigarettes for cigarettes.^2^ However, it is also important to note that dual use of cigarettes and e-cigarettes has increased over time^24^ and that among people who dual use cigarettes and e-cigarettes, most individuals smoke cigarettes more frequently than they use e-cigarettes.^25^ Furthermore, individuals who dual use remain exposed to cigarette smoking toxicants,^26,27^ making complete rather than partial substitution of cigarettes with e-cigarettes important for risk reduction.^28^ Indeed, health care clinicians are now encouraged to discuss e-cigarettes as a cessation tool with patients who smoke cigarettes and have already tried FDA-approved cessation medications.^29^

We also found that cigarette cessation rates were higher for adults who used e-cigarettes in 2019 to 2021 compared with those who used e-cigarettes before 2016 even after controlling for e-cigarette use frequency. This finding is consistent with the hypothesis that newer-generation e-cigarettes that contain salt-based nicotine formulations with improved nicotine delivery are more effective for smoking cessation.^30^ However, other factors may have contributed to the increased cessation rates observed across our study period; these factors may include the COVID-19 pandemic, with prior findings on the association of the COVID-19 pandemic with smoking cessation being mixed.^31,32,33^ Additionally, the PATH Study 2021 data collection used telephone and in-person interviews,^12^ which differed from earlier years and may have impacted estimated cigarette cessation rates, although we controlled for PATH Study protocol differences in adjusted analyses.

The CTP has acted on more than 99% of e-cigarette premarket tobacco product applications that were submitted, and thus far has authorized only tobacco flavor e-cigarettes to be sold as meeting the standard for the protection of public health.^34^ We found associations between e-cigarette flavor (menthol or mint vs tobacco flavor) and cessation outcomes to be sensitive to the timing of assessment of e-cigarette use and cigarette cessation; use of menthol or mint flavor e-cigarettes vs tobacco flavor e-cigarettes at baseline (ie, 1-2 years prior to outcome assessment) was associated with overall smoking discontinuation at follow-up even after controlling for other variables; however, e-cigarette flavor was not associated with cigarette discontinuation when e-cigarette use was assessed at follow-up alongside assessment of the outcome. Exploratory interaction analyses among covariates did not yield explanations for these findings, although statistical power was diminished. An untested potential explanation may be differences in changes in e-cigarette use frequency over time for different flavors.

It is also possible that menthol or mint flavor findings may be an artifact of CTP’s 2020 enforcement prioritization against nontobacco- and nonmenthol-flavored, cartridge-based e-cigarette products.^35^ Nielsen Retail Scanner data showed a 43% increase in market share of menthol-flavored e-cigarettes over 4 weeks after CTP’s 2020 e-cigarette enforcement priorities.^36^ Additionally, given that people who smoke menthol cigarettes tend to smoke fewer cigarettes than people who smoke nonmenthol cigarettes,^37^ there may be underlying interactions of cigarette smoking frequency with cigarette or e-cigarette flavor use and e-cigarette use frequency with cigarette or e-cigarette flavor use. Subsequent research limited to e-cigarettes marketed in recent years to reduce potential confounding of time period effects may be able to disentangle cross-product flavor and cross-product use frequency congruence and incongruence.

E-cigarette device type was not associated with any cigarette cessation outcome after controlling for e-cigarette use frequency, year of data collection, and other variables. However, assessment of e-cigarette device type was aggregated across time, and early-generation disposable e-cigarettes (cigalikes) were very different from disposable e-cigarettes marketed in recent years. Further research is needed to determine whether the association between e-cigarette device type and cigarette cessation has changed over time. Given that cigarette cessation rates were higher among adults who used e-cigarettes in recent times vs earlier times, research focused on e-cigarettes marketed in recent years is important to addressing questions such as whether today’s disposable e-cigarettes differ from today’s tank e-cigarettes in their associations with cigarette cessation.

### Limitations

This study has several limitations. We were not able to consider e-cigarette flavor and device use between waves, and the 2 approaches to the temporal ordering of e-cigarette use relative to outcome assessment have limitations; e-cigarette use assessed at baseline yields an analytic sample of respondents who dual used cigarettes and e-cigarettes, excluding those who may have used e-cigarettes and had already quit smoking by baseline (ie, those who formerly smoked cigarettes). However, e-cigarette use assessed at follow-up excludes respondents who may have used e-cigarettes, quit smoking, and no longer used e-cigarettes at follow-up, or it may itself be a marker for cigarette cessation success. Relatedly, frequent e-cigarette use may be an indicator of the process of quitting rather than an independent factor associated with quitting. Such a distinction is important to consider from policy and practice standpoints, where promoting daily use of e-cigarettes may not be effective in encouraging quitting if daily use is part of the process of quitting.

We were unable to directly evaluate the association between e-cigarette nicotine formulation and cigarette cessation outcomes, and year of data collection as a proxy for this measure was confounded with the COVID-19 pandemic and enactment of national and subnational tobacco control policies.^34,38^ Additionally, our study evaluated only adults who smoked cigarettes and used e-cigarettes and does not speak to overall effectiveness of e-cigarette use for smoking cessation.

## Conclusions

In this cohort study of adults in the US population who used e-cigarettes, daily e-cigarette use and use of e-cigarettes in 2019 to 2021 were consistently associated with greater cigarette discontinuation rates. Menthol or mint flavor vs tobacco flavor e-cigarette use among adults who dual used cigarettes and e-cigarettes was associated with more than 2-fold higher odds of cigarette discontinuation 1 to 2 years later. Further research is needed to determine whether access to menthol or mint flavor e-cigarettes for adults who smoke cigarettes daily may be important for the protection of public health. Our findings suggest that e-cigarette public health decisions should be based on data from e-cigarettes marketed in recent years that have evolved outside the regulated market and that randomized clinical trials are warranted using these products.

## Notes

Supplement 1.
eMethods.
**eFigure 1.** First Approach to Analysis **eFigure 2.** Second Approach to Analysis **eTable 1.** Population of Adults Ages ≥21 y Who Smoked Cigarettes at Baseline and Used E-Cigarettes at Follow-Up **eTable 2.** Making a Quit Attempt at Follow-Up by E-Cigarette Characteristics **eTable 3.** Cigarette Cessation at Follow-Up Among Adults Who Made a Cigarette Quit Attempt by E-Cigarette Characteristics **eTable 4.** Discontinuing Past 30-day Cigarette Smoking by E-Cigarette Characteristics

Supplement 2.
Data Sharing Statement
