# Supplementary material for: E-Cigarette Characteristics and Cigarette Cessation Among Adults Who Use E-Cigarettes
Source: JAMA Netw Open. 2024 Aug 1;7(8):e2423960. doi: 10.1001/jamanetworkopen.2024.23960 (PMC11294961; doi:10.1001/jamanetworkopen.2024.23960)
Supplement: Supplement 1. — eMethods. eFigure 1. First Approach to Analysis eFigure 2. Second Approach to Analysis eTable 1. Population of Adults Ages ≥21 y Who Smoked Cigarettes at Baseline and Used E-Cigarettes at Follow-Up eTable 2. Making a Quit Attempt at Follow-Up by E-Cigarette Characteristics eTable 3. Cigarette Cessation at Follow-Up Among Adults Who Made a Cigarette Quit Attempt by E-Cigarette Characteristics eTable 4. Discontinuing Past 30-day Cigarette Smoking by E-Cigarette Characteristics [file jamanetwopen-e2423960-s001.pdf]

Supplemental Online Content

Kasza KA, Rivard C, Goniewicz ML, et al. E-cigarette characteristics and cigarette cessation among adults who use e-cigarettes. *JAMA Netw Open*. 2024;7(8):e2423960. doi:10.1001/jamanetworkopen.2024.23960

eMethods.

eFigure 1. First approach to analysis

eFigure 2. Second approach to analysis

eTable 1. Population of adults ages ≥21 y who smoked cigarettes at baseline and used e-cigarettes at follow-up

eTable 2. Making a quit attempt at follow-up by e-cigarette characteristics

eTable 3. Cigarette cessation at follow-up among adults who made a cigarette quit attempt by e-cigarette characteristics

eTable 4. Discontinuing past 30-day cigarette smoking by e-cigarette characteristics

This supplemental material has been provided by the authors to give readers additional information about their work.

© 2024 Kasza KA et al. *JAMA Network Open.*

eMethods

Analysis approaches: The first approach to analysis is depicted in eFigure 1; we conducted analyses among adults ages 21+ years who smoked cigarettes daily at baseline wave of a wave pair and who used e-cigarettes in the past 30 days at baseline wave of the wave pair (n=1,985, eFigure 1). The second approach to analysis is depicted in eFigure 2; we conducted analyses among those ages 21+ years who smoked cigarettes daily at baseline and used e-cigarettes in the past 30 days at follow-up (n=2,308, eFigure 2). The first approach excludes from the sample those who used e-cigarettes and already quit smoking at baseline, whereas the second approach does not distinguish between whether e-cigarette use preceded or followed cigarette cessation. Also, the most recent e-cigarette use data used in the first approach is necessarily drawn from 2018/19 whereas the most recent e-cigarette use data used in the second approach is necessarily drawn from 2021.

© 2024 Kasza KA et al. *JAMA Network Open.*

eFigure 1. First approach to analysis: Sample includes adults ages 21+ years who smoked cigarettes daily at baseline and who used e-cigarettes in the past 30 days at baseline. Cigarette cessation behaviors assessed at follow-up.

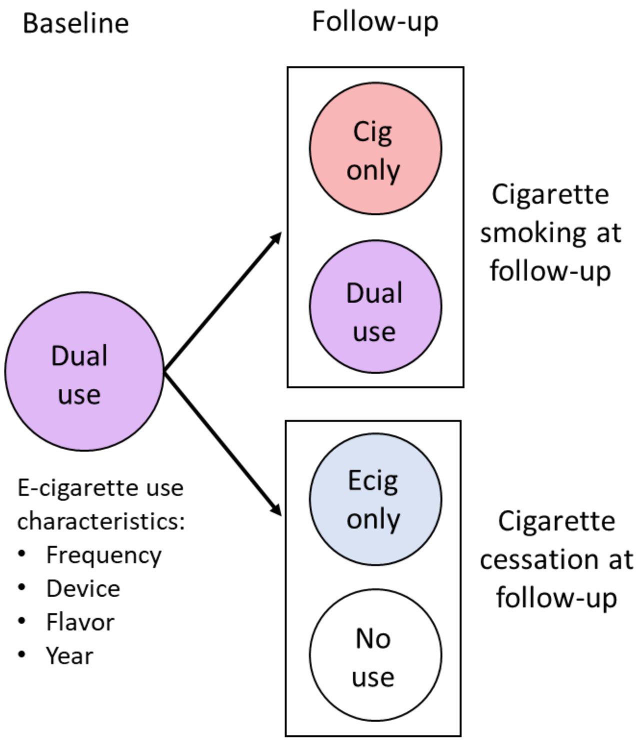

Note. This approach excludes from the sample those who used e-cigarettes and already quit smoking at baseline.

eFigure 2. Second approach to analysis: Sample includes adults ages 21+ years who smoked cigarettes daily at baseline and who used e-cigarettes in the past 30 days at follow-up. Cigarette cessation behaviors assessed at follow-up.

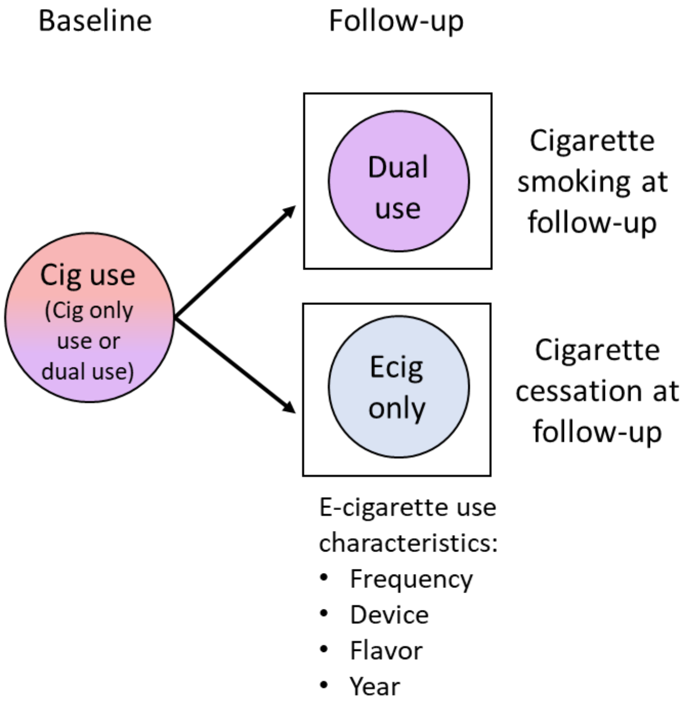

Note. This approach does not distinguish between whether e-cigarette use preceded or followed cigarette cessation.

© 2024 Kasza KA et al. *JAMA Network Open.*

eTable 1. Composition of the population of adults ages 21+ years who smoked cigarettes at baseline and used e-cigarettes at follow-up.

	n	%	95%CI
**Age**			
(mean)	2308	38.5	37.7-39.3
**Sex**			
Male	965	49.9	46.4-53.4
Female	1343	50.1	46.6-53.6
**Race**			
Black	295	10.9	9.3-12.6
White	1771	80.9	78.2-83.3
Other race including multiple races[1]	242	8.2	6.5-10.3
**Ethnicity**			
Hispanic	250	9.4	7.7-11.3
Non-Hispanic	2058	90.6	88.7-92.3
**Cigarettes smoked per day (CPD)**			
1-9 CPD	648	25.5	23.1-28.0
10-19 CPD	849	35.9	33.2-38.6
20+ CPD	811	38.7	35.8-41.6
**Menthol/nonmenthol cigarette smoking**			
Menthol	1128	46.5	43.5-49.6
Nonmenthol	1160	52.8	49.7-55.8
Unknown/missing	20	0.7	0.4-1.2
**E-cigarette use frequency**			
Daily	1718	72.5	70.0-74.8
Nondaily	590	27.5	25.2-30.0
**E-cigarette device type used**			
Disposable	506	21.6	19.1-24.2
Cartridge	792	33.4	30.9-35.9
Tank	1010	45.1	42.3-47.9
**E-cigarette flavor(s) used**			
Only tobacco flavor	531	24.7	22.3-27.4
Only menthol/mint flavor	413	16.8	14.9-18.8
Only sweet flavor(s)	1132	48.1	45.4-50.7
Any combination of tobacco, menthol/mint, sweet flavor(s)	232	10.4	8.7-12.3
**Year of data collection**			
2014/15-2015/16 (W2-W3)	609	27.4	25.5-29.4
2015/16-2017 (W3-W4)	514	22.2	20.5-23.9
2017-2019 (W4-W5)	661	28.7	27.1-30.3
2019-2021 (W5-W6)	524	21.7	20.0-23.6

eTable 1 notes.

Sample sizes are unweighted; %s and 95%CIs are weighted.

[1]Other race including multiple races includes American Indian or Alaska Native, Asian Indian, Chinese, Filipino, Japanese, Korean, Vietnamese, Other Asian, Native Hawaiian, Guamanian or Chamorro, Samoan, Other Pacific Islander.

© 2024 Kasza KA et al. *JAMA Network Open.*

eTable 2: Making a quit attempt at follow-up among adults (ages 21+) who smoked cigarettes daily at baseline and used e-cigarettes in the past 30 days at follow-up, as a function of e-cigarette characteristics.

Population: Adults who smoked cigarettes daily at baseline and used e-cigarettes in past 30 days at follow-up		**Cigarette quit attempt at follow-up** (i.e., Daily smoking at baseline → Made a quit attempt at follow-up)								
					**Model 1[1]**			**Model 2[2]**		
**E-cigarette characteristics (n=2,195)**		n	%	95% CI	AOR[1]	95% CI		AOR[2]	95% CI	
**Frequency of e-cigarette use**	Nondaily (n=1,608)	646	39.0	[36.2,41.9]	NA			1.00	Referent	
	Daily (n=587)	400	66.6	[62.0,71.0]				**3.12*** **	**2.42**	**4.02**
**Device type used**	(1) Disposable (n=471)	198	42.0	[37.1,47.1]	1.00	Referent		1.00	Referent	
	(2) Cartridge (n=747)	348	44.5	[40.1,49.0]	1.20	0.88	1.63	1.11	0.81	1.51
	(3) Tank/mod (n=977)	500	51.1	[47.1,55.0]	**1.50***	**1.05**	**2.14**	1.24	0.88	1.75
**Flavor(s) used**	(1) Only tobacco flavor (n=497)	208	40.4	[35.3,45.7]	1.00	Referent		1.00	Referent	
	(2) Only menthol/mint flavor (n=386)	182	46.4	[41.0,51.9]	1.18	0.79	1.77	1.09	0.72	1.63
	(3) Only sweet flavor(s) (n=1,087)	550	49.8	[46.1,53.5]	1.21	0.90	1.62	1.09	0.80	1.48
	(4) Any combination of tobacco, menthol/mint, sweet flavor(s) (n=225)	106	50.5	[42.4,58.6]	1.28	0.84	1.94	1.12	0.72	1.73
**Year of Data Collection**	2014/15-2015/16 (W2-W3) (n=505)	230	45.9	[40.7,51.1]	1.00	Referent		1.00	Referent	
	2015/16-2017 (W3-W4) (n=507)	232	45.9	[40.7,51.2]	1.01	0.73	1.40	1.05	0.77	1.44
	2017-2019 (W4-W5) (n=659)	309	45.5	[40.8,50.3]	1.00	0.74	1.37	1.00	0.73	1.37
	2019-2021 (W5-W6) (n=524)	275	51.3	[46.4,56.1]	1.27	0.88	1.83	1.11	0.75	1.66

eTable 2 notes.

Ns indicate unweighted numbers of observations; percentages, Adjusted Odds Ratios (AOR) and 95% Confidence Intervals (CIs) are weighted using the Wave 6 all-waves weights for the Wave 1 cohort.

GEE logistic regression analyses were used to assess associations between e-cigarette characteristics and making a cigarette quit attempt using four wave pairs (i.e., W2-W3, W3-W4, W4-W5, W5-W6), including up to four change data points per individual and statistically controlling for the correlation among observations from the same individuals.

[1]Model 1 adjusts for sex, race, ethnicity, age, cigarettes smoked per day, menthol/nonmenthol cigarette smoking, interview mode, e-cigarette device type (except when evaluating the main effect of e-cigarette device type used), e-cigarette flavor(s) (except when evaluating the main effect of e-cigarette flavor(s) used), and year of data collection (except when evaluating the main effect of year of data collection).

[2]Model 2 adjusts for the same covariates as in Model 1 plus e-cigarette use frequency (except when evaluating the main effect of e-cigarette use frequency).

*p<0.05; **p<.01; ***p<.001

© 2024 Kasza KA et al. *JAMA Network Open.*

eTable 3: Cigarette cessation at follow-up among adults (ages 21+) who smoked cigarettes daily at baseline, used e-cigarettes in the past 30 days at follow-up, and made a cigarette quit attempt between baseline and follow-up, as a function of e-cigarette characteristics.

Population: Adults who smoked cigarettes daily at baseline, used e-cigarettes in past 30 days at follow-up, and made a cigarette quit attempt between baseline and follow-up		**Cigarette cessation at follow-up** (i.e., Daily smoking at baseline, made a quit attempt between baseline and follow-up → No daily/nondaily smoking at follow-up)								
					**Model 1[1]**			**Model 2[2]**		
**E-cigarette characteristics (n=1,046)**		n	%	95% CI	AOR[1]	95% CI		AOR[2]	95% CI	
**Frequency of e-cigarette use**	Nondaily (n=646)	87	15.2	[11.9,19.3]	NA			1.00	Referent	
	Daily (n=400)	216	52.8	[46.4,59.2]				**6.09*****	**3.89**	**9.54**
**Device type used**	(1) Disposable (n=198)	60	30.4	[22.8,39.3]	1.00	Referent		1.00	Referent	
	(2) Cartridge (n=348)	91	29.2	[23.0,36.3]	1.30	0.76	2.22	1.05	0.56	1.96
	(3) Tank/mod (n=500)	152	31.7	[27.0,36.7]	1.42	0.82	2.47	0.88	0.48	1.62
**Flavor(s) used**	(1) Only tobacco flavor (n=208)	44	24.1	[16.8,33.4]	1.00	Referent		1.00	Referent	
	(2) Only menthol/mint flavor (n=182)	44	27.9	[20.4,36.8]	1.07	0.51	2.24	0.87	0.41	1.88
	(3) Only sweet flavor(s) (n=550)	185	35.3	[30.6,40.2]	1.48	0.83	2.65	1.38	0.78	2.42
	(4) Any combination of tobacco, menthol/mint, sweet flavor(s) (n=106)	30	25.8	[16.8,37.6]	0.96	0.45	2.02	0.81	0.34	1.93
**Time period**	2014/15-2015/16 (W2-W3) (n=230)	59	27.3	[20.7,35.1]	1.00	Referent		1.00	Referent	
	2015/16-2017 (W3-W4) (n=232)	41	17.0	[12.9,22.1]	**0.57***	**0.33**	**0.99**	0.63	0.34	1.15
	2017-2019 (W4-W5) (n=309)	78	29.3	[23.1,36.4]	1.09	0.64	1.86	1.13	0.65	1.98
	2019-2021 (W5-W6) (n=275)	125	47.5	[39.7,55.4]	**2.65****	**1.45**	**4.85**	**2.16***	**1.13**	**4.11**

eTable 3 notes.

Ns indicate unweighted numbers of observations; percentages, Adjusted Odds Ratios (AOR) and 95% Confidence Intervals (CIs) are weighted using the Wave 6 all-waves weights for the Wave 1 cohort.

GEE logistic regression analyses were used to assess associations between e-cigarette use characteristics and cigarette cessation among those who made a quit attempt using four wave pairs (i.e., W2-W3, W3-W4, W4-W5, W5-W6), including up to four change data points per individual and statistically controlling for the correlation among observations from the same individuals.

[1]Model 1 adjusts for sex, race, ethnicity, age, cigarettes smoked per day, menthol/nonmenthol cigarette smoking, interview mode, e-cigarette device type (except when evaluating the main effect of e-cigarette device type used), e-cigarette flavor(s) (except when evaluating the main effect of e-cigarette flavor(s) used), and year of data collection (except when evaluating the main effect of year of data collection).

[2]Model 2 adjusts for the same covariates as in Model 1 plus e-cigarette use frequency (except when evaluating the main effect of e-cigarette use frequency).

*p<0.05; **p<.01; ***p<.001

© 2024 Kasza KA et al. *JAMA Network Open.*

eTable 4: Discontinuing past 30-day cigarette smoking at follow-up among adults (ages 21+) who smoked cigarettes daily at baseline and used e-cigarettes in the past 30 days at follow-up, as a function of e-cigarette characteristics.

Population: Adults who smoked cigarettes daily at baseline and used e-cigarettes in the past 30 days at follow-up		**Discontinue cigarette smoking at follow-up** (i.e., Daily smoking at baseline → No past 30-day smoking at follow-up)								
					**Model 1[1]**			**Model 2[2]**		
**E-cigarette characteristics (n=2,308)**		n	%	95% CI	AOR[1]	95% CI		AOR[2]	95% CI	
**Frequency of e-cigarette use**	Nondaily (n=1,718)	53	3.0	[2.2,4.1]	NA			1.00	Referent	
	Daily (n=590)	171	27.8	[22.7,33.6]				**11.20*** **	**6.92**	**18.12**
**Device type used**	(1) Disposable (n=506)	44	8.1	[5.7,11.4]	1.00	Referent		1.00	Referent	
	(2) Cartridge (n=792)	69	8.6	[6.3,11.7]	**1.85***	**1.11**	**3.10**	1.53	0.86	2.71
	(3) Tank/mod (n=1,010)	111	11.6	[9.1,14.5]	**2.06** **	**1.29**	**3.28**	1.34	0.80	2.23
**Flavor(s) used**	(1) Only tobacco flavor (n=531)	30	5.8	[3.6,9.3]	1.00	Referent		1.00	Referent	
	(2) Only menthol/mint flavor (n=413)	30	7.6	[5.0,11.2]	1.06	0.54	2.06	0.85	0.43	1.69
	(3) Only sweet flavor(s) (n=1,132)	141	12.7	[10.2,15.6]	**1.91***	**1.02**	**3.57**	1.69	0.85	3.36
	(4) Any combination of tobacco, menthol/mint, sweet flavor(s) (n=232)	23	10.1	[6.1,16.2]	1.63	0.75	3.53	1.24	0.49	3.17
**Year of Data Collection**	2014/15-2015/16 (W2-W3) (n=609)	33	6.0	[4.1,8.7]	1.00	Referent		1.00	Referent	
	2015/16-2017 (W3-W4) (n=514)	28	4.6	[3.1,6.7]	0.72	0.39	1.33	0.71	0.37	1.37
	2017-2019 (W4-W5) (n=661)	55	9.4	[7.0,12.6]	1.55	0.93	2.59	1.45	0.86	2.45
	2019-2021 (W5-W6) (n=524)	108	20.7	[16.4,25.7]	**4.96*** **	**3.06**	**8.03**	**4.17*** **	**2.48**	**7.00**

eTable 4 notes.

Ns indicate unweighted numbers of observations; percentages, Adjusted Odds Ratios (AOR) and 95% Confidence Intervals (CIs) are weighted using the Wave 6 all-waves weights for the Wave 1 cohort.

GEE logistic regression analyses were used to assess associations between e-cigarette use characteristics and discontinuing cigarette smoking using four wave pairs (i.e., W2-W3, W3-W4, W4-W5, W5-W6), including up to four change data points per individual and statistically controlling for the correlation among observations from the same individuals.

[1]Model 1 adjusts for sex, race, ethnicity, age, cigarettes smoked per day, menthol/nonmenthol cigarette smoking, interview mode, e-cigarette device type (except when evaluating the main effect of e-cigarette device type used), e-cigarette flavor(s) (except when evaluating the main effect of e-cigarette flavor(s) used), and year of data collection (except when evaluating the main effect of year of data collection).

[2]Model 2 adjusts for the same covariates as in Model 1 plus e-cigarette use frequency (except when evaluating the main effect of e-cigarette use frequency).

*p<0.05; **p<.01; ***p<.001

© 2024 Kasza KA et al. *JAMA Network Open.*
