# Supplementary material for: E-Cigarette Characteristics and Cigarette Cessation Among Adults Who Use E-Cigarettes
Source: JAMA Netw Open. 2024 Aug 1;7(8):e2423960. doi: 10.1001/jamanetworkopen.2024.23960 (PMC11294961; doi:10.1001/jamanetworkopen.2024.23960)
Supplement: Supplement 2. — Data Sharing Statement [file jamanetwopen-e2423960-s002.pdf]

Data Sharing Statement

Kasza. E-Cigarette Characteristics and Cigarette Cessation Among Adults Who Use E-Cigarettes. *JAMA Netw Open*. Published online August 1, 2024. doi:10.1001/jamanetworkopen.2024.23960

Data
Data available: Yes
Data types: Deidentified participant data, Data dictionary
How to access data: Data from the PATH Study may be obtained from a third party and are not publicly available (https://www.icpsr.umich.edu/icpsrweb/NAHDAP/studies/36231). Application instructions and conditions of use are available at the website previously mentioned.
When available: With publication

Supporting Documents
Document types: None

Additional Information
Who can access the data: Data from the PATH Study may be obtained from a third party and are not publicly available (https://www.icpsr.umich.edu/icpsrweb/NAHDAP/studies/36231). Application instructions and conditions of use are available at the website previously mentioned.
Types of analyses: Data from the PATH Study may be obtained from a third party and are not publicly available (https://www.icpsr.umich.edu/icpsrweb/NAHDAP/studies/36231). Application instructions and conditions of use are available at the website previously mentioned.
Mechanisms of data availability: Data from the PATH Study may be obtained from a third party and are not publicly available (https://www.icpsr.umich.edu/icpsrweb/NAHDAP/studies/36231). Application instructions and conditions of use are available at the website previously mentioned.
